# Port-Site Metastasis After Laparoscopic Gastrectomy Extending to the Thigh: A Case Report

**DOI:** 10.7759/cureus.60273

**Published:** 2024-05-14

**Authors:** Kentaro Goto, Yasutaka Nakanishi, Masashi Saji, Hiroaki Hata

**Affiliations:** 1 Department of Surgery, National Hospital Organization Kyoto Medical Center, Kyoto, JPN; 2 Division of Gastrointestinal Surgery, Department of Surgery, Kyoto University, Kyoto, JPN

**Keywords:** abdominal wall recurrence, gastric cancer, laparoscopic surgery, muscle metastasis, port-site recurrence

## Abstract

Port-site metastasis (PSM) is rare following laparoscopic gastrectomy for gastric cancer. Previous reports focused on localized lesions treated with excision; contrastingly, case reports describing extensive invasion into the lower extremity skeletal muscles causing deterioration in activities of daily living are nonexistent. A 55-year-old male underwent a laparoscopic distal gastrectomy for gastric cancer. The pathological findings revealed a stage IIIA tumor. Two years later, skin hardening was observed on the left upper abdominal wall. Computed tomography displayed a 13-cm-long, flat tumor along the skeletal muscle around the left upper 12 mm port site and right hydronephrosis. The patient was diagnosed with PSM and retroperitoneal recurrence. Despite chemotherapy, three years postoperatively, PSM widely spread from the left upper abdomen to the left thigh, eventually inducing opioid-resistant leg pain and subsequent walking difficulties. Palliative radiotherapy could not improve these symptoms. The patient died three years and five months postoperatively. Extensively invasive PSM can induce refractory cancer pain and physical disorders. Therefore, early detection and palliative resection of PSM may help maintain the quality of life of patients with gastric cancer.

## Introduction

Gastric cancer is the fifth-most common cancer in the world [1]. Laparoscopic gastrectomy for curative resection is a popular procedure for both early and advanced gastric cancer [2]. Port-site metastasis (PSM) following laparoscopic gastrectomy is rare and has been sporadically reported [3-7]. These reports only documented resected cases for localized PSM; however, these reports have not clarified the clinicopathological features of gastric cancer and their association with the occurrence of PSM, and there are no reports of PSM extending into the skeletal muscles and deteriorating activities of daily living (ADL). We report a case of PSM after gastrectomy, which proliferated along the abdominal wall to the thigh, resulting in pain and difficulty in walking.

## Case presentation

**Figure 1:**
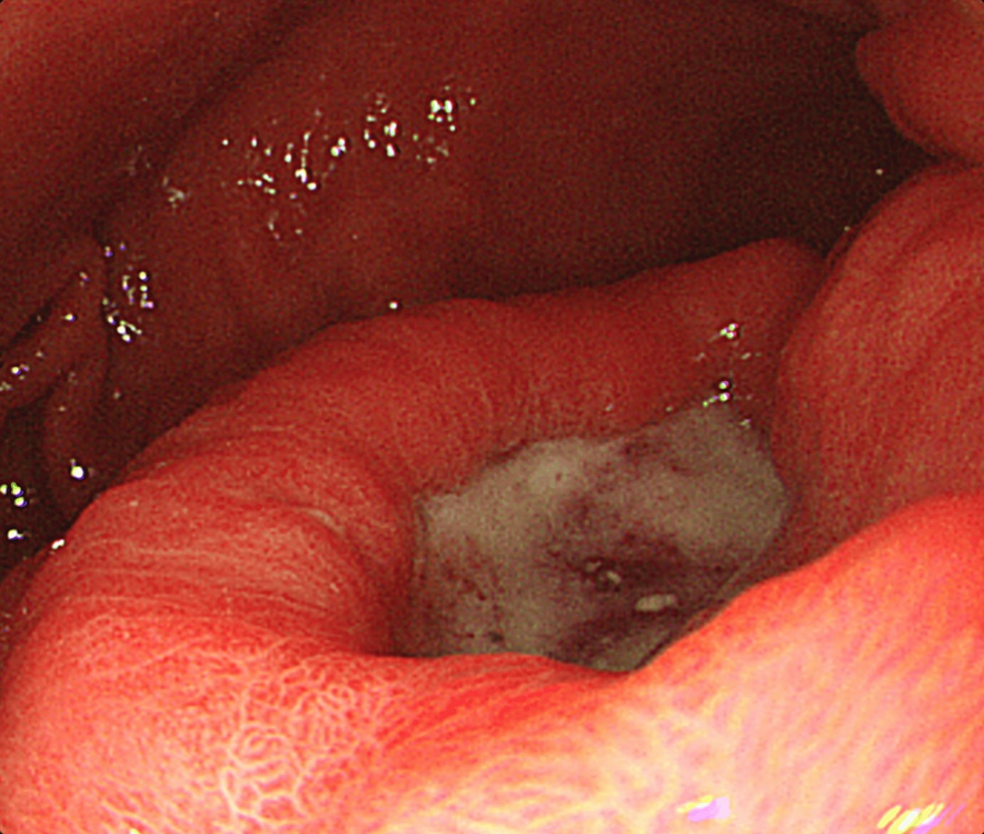
Endoscopic findings of the primary tumor.

**Figure 2:**
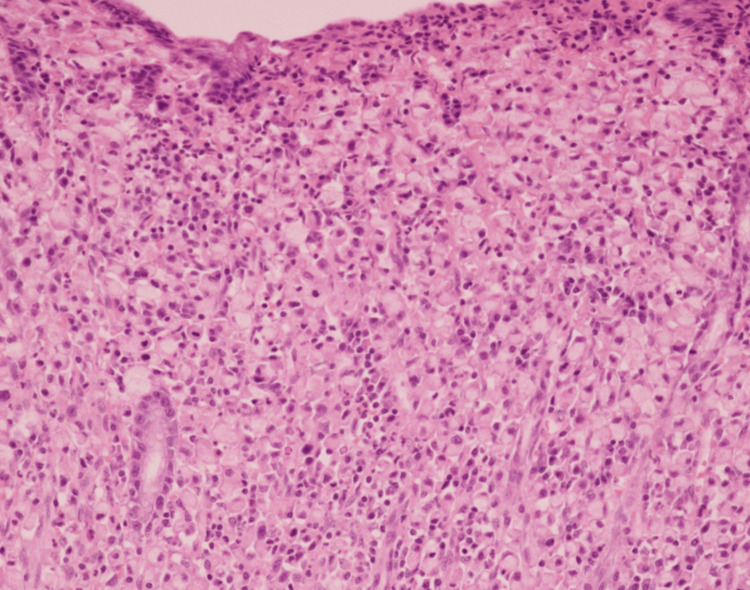
Histological findings from hematoxylin-eosin-stained specimen.

**Figure 3:**
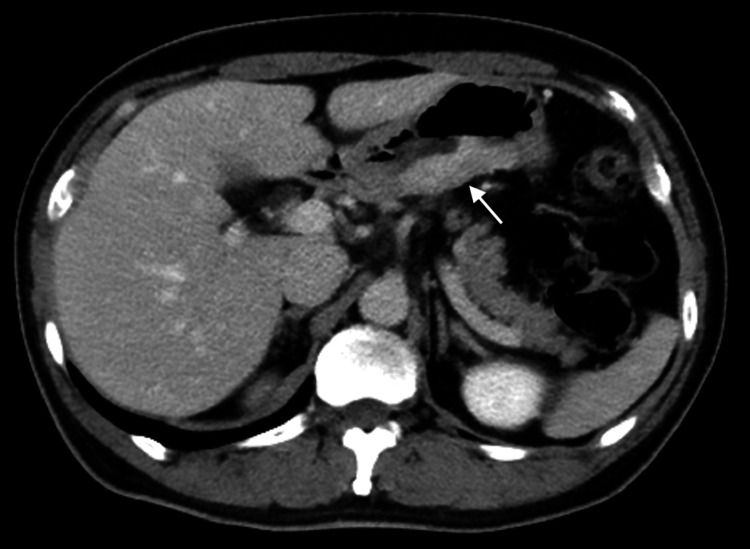
Preoperative contrast-enhanced computed tomography. The tumor is shown as a contrast-enhanced lesion in the posterior gastric wall (white arrow).

**Figure 4:**
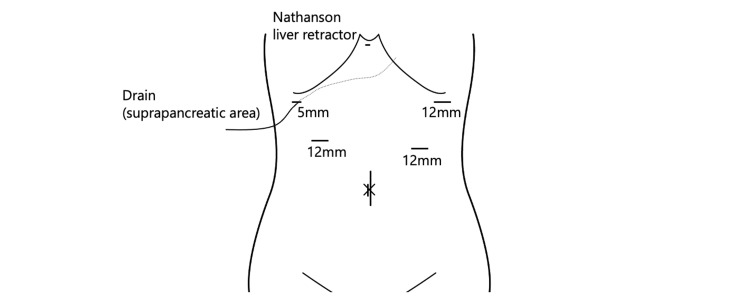
Trocar placement at surgery. The umbilical incision is extended for specimen retrieval. An Information drain is placed at the suprapancreatic region from the upper right 5 mm port. Image created by the authors.

A 55-year-old male was referred to our hospital for treatment of gastric cancer associated with upper abdominal pain. Upper gastrointestinal endoscopy revealed a type II tumor on the posterior wall of the antrum (Figure 1). Histological examination revealed poorly differentiated adenocarcinoma with signet ring cells on biopsy (Figure 2). Contrast-enhanced computed tomography (CT) did not detect lymph node enlargement and distant metastases (Figure 3). The patient was diagnosed with advanced gastric cancer at clinical stage IIB, T4a N0 M0, according to the UICC 8th edition staging system. According to the Japanese Gastric Cancer Treatment Guidelines (6th edition), a laparoscopic radical D2 distal gastrectomy was performed. After inserting the camera through the umbilical port, we confirmed the absence of peritoneal dissemination. The trocars were placed as shown in Figure 4, and a Nathanson liver retractor was inserted through the epigastric region. Peritoneal washing cytology was negative. The surgeon positioned on the right side except for the #6 lymph nodes (LNs) dissection. The gauze used for hemostat and traction on peripancreatic tissue was exchanged through each 12 mm port. The resected specimen was removed through an extended umbilical port incision using a specimen retrieval bag. Subsequently, an antecolic Roux-en-Y reconstruction was performed intracorporeally. An information drain (Jackson-Pratt-type flat drain) was placed in the suprapancreatic region from the right upper 5 mm port. The operating time was 271 minutes, and the estimated blood loss was 30 ml. The postoperative course was uneventful, and the drain was removed on the second postoperative day. The patient was discharged on postoperative day 12.

**Figure 5:**
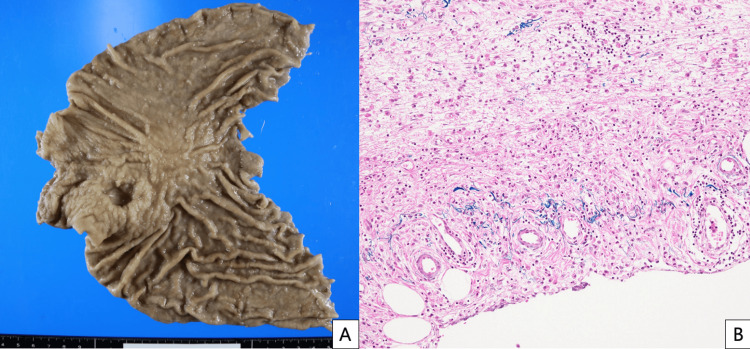
Pathological findings of resected specimen. A: macroscopic findings; B: microscopic findings

**Figure 6:**
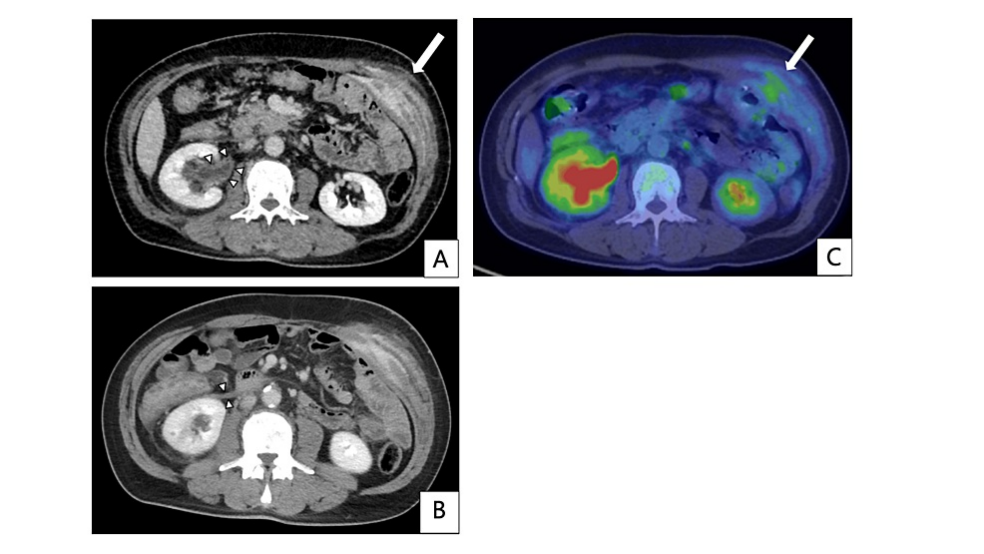
Contrast-enhanced computed tomography and positron emission tomography-computed tomography (PET-CT) at the time of port-site recurrence, taken two years postoperatively. A, B: A flat, contrast-enhancing tumor measuring 13 cm in maximum diameter is identified in the subcutaneous tissue, external/internal oblique muscle, and transversus abdominis muscles around the left upper abdominal 12 mm port site (indicated by the white arrow). Concurrent thickening of the right renal pelvis wall (white arrowheads in A), as well as right hydronephrosis and thickening of the anterior renal fascia (white arrowheads in B), are also noted. C: PET-CT showing an accumulation with a max-standardized uptake value (max-SUV) of 3.68 in the abdominal wall lesion.

The pathological diagnosis of the resected specimen was stage IIIA; pT4a (SE), N1 (1/41, metastatic lymph node station #6), M0, with negative proximal and distal margins (Figure 5). A venous invasion was present, but no lymphatic invasion was recognized. The patient underwent adjuvant chemotherapy with S-1 for one year after the operation. There was no recurrence sign in CT follow-up at one year and six months postoperatively. Two years postoperatively, induration was observed at the left upper abdomen without any changes on the skin surface. Contrast-enhanced CT revealed a 13-cm-long flat tumor recognized as a contrast-enhancing lesion in the skeletal muscle around the left-upper 12 mm port site. Thickening of the right renal pelvis and ureter wall, as well as right hydronephrosis, were also detected (Figure 6). Positron emission tomography-computed tomography (PET-CT) showed an accumulation with a max-standardized uptake value (max-SUV) of 3.68 in the abdominal wall tumor. The patient was diagnosed with retroperitoneal and port-site recurrence and treated with S-1 plus cisplatin chemotherapy.

**Figure 7:**
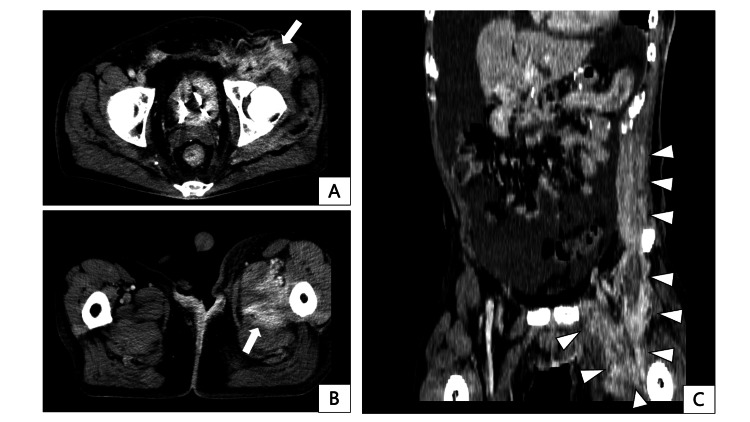
Contrast-enhanced computed tomography at the time of port-site metastasis extension to the left thigh, taken three years postoperatively. A, B: Axial images. High-density areas with contrast enhancement are observed subcutaneously at the left inguinal region, extending to the left thigh quadriceps and adductor muscles (indicated by the white arrow). C: Coronal image. High-density areas in the left inguinal subcutaneous and thigh muscles appear continuous with the known left abdominal wall port-site metastasis (indicated by the white arrowheads).

**Figure 8:**
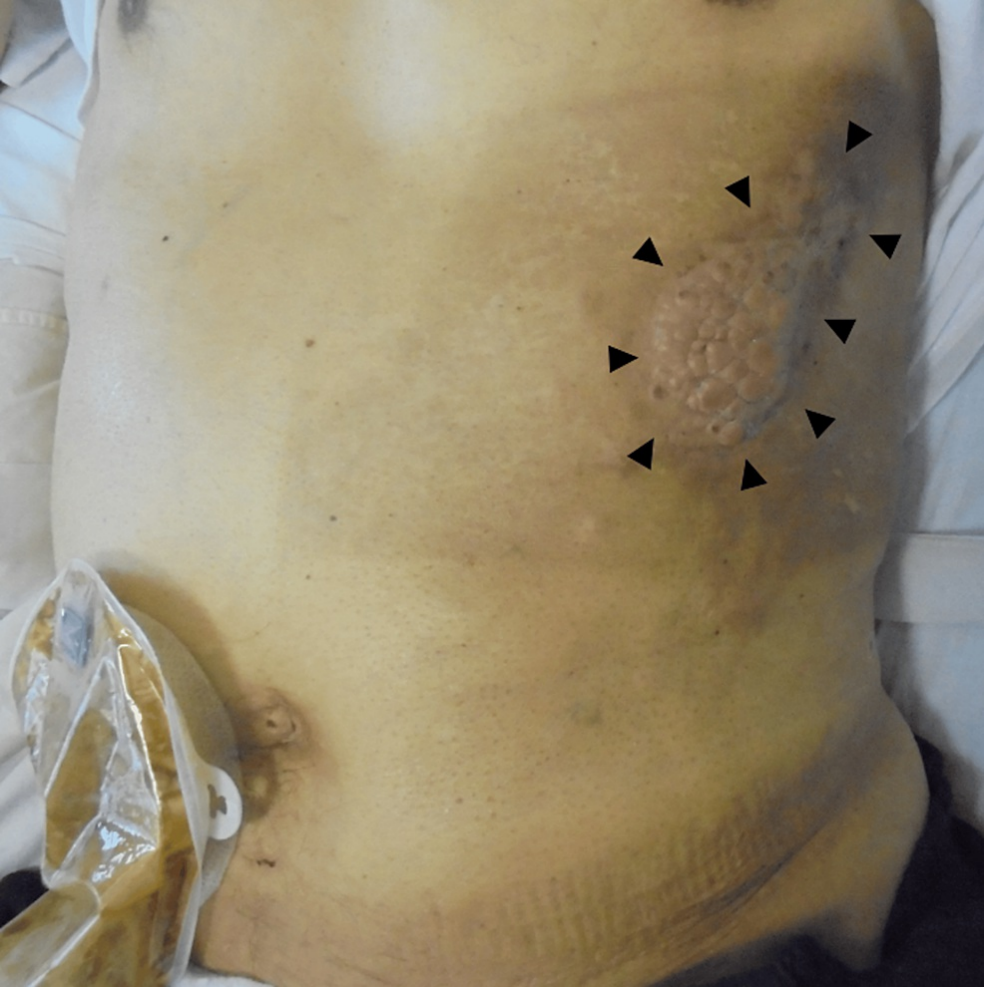
Photograph of the skin infiltration from port-site metastasis (indicated by the black arrowheads). The lesion had a hyperpigmented area of nodular aspect.

With two years and seven months postoperative CT, the patient was diagnosed with intestinal obstruction in the transverse colon and rectum due to peritoneal dissemination, necessitating an ileostomy. Although paclitaxel was initiated as second-line chemotherapy, three years postoperatively, contrast-enhanced CT demonstrated longitudinal extension of the port-site recurrence to the left inguinal region, quadriceps, and adductor muscles in the left thigh (Figure 7). Nivolumab was administered as a third-line chemotherapy. At three years and one month postoperatively, there was enlargement of the skin infiltration at the PSM (Figure 8). Radiotherapy of 30 Gy in 10 fractions was administered to prevent spontaneous skin ulceration at the PSM, and irinotecan was started as fourth-line chemotherapy. Due to the enlargement of the inguinal lesion, the patient developed opioid-resistant inguinal pain, leading to walking difficulties. Radiotherapy of 20 Gy in five fractions was administered to the inguinal lesion to alleviate cancer pain. The skin-invading lesion slightly decreased in thickness with radiotherapy but developed regrowth two months after irradiation. Neither pain nor ADL could be improved for the inguinal lesion. At three years and four months postoperatively, the patient developed obstructive jaundice due to peritoneal dissemination at the hepatic hilum and difficulty in alleviating jaundice that eventually led to the best supportive care. The patient died three years and five months postoperatively.

## Discussion

PSM after gastric cancer surgery is considered rare compared to other cancers [8]. The incidence of PSM is reported to be 0.9%-2.4% for colorectal cancer [9,10], 6.7% for biliary tract cancer (mainly cholecystectomy for unsuspected gallbladder carcinoma) [11], 2.3% for gynecological cancer [12], and 0.18% for urological cancer [13]. Although the reported incidence rate of PSM following exploratory laparoscopy for upper gastrointestinal tract malignancies is 0.79% [14], a retrospective cohort study of 601 patients has reported no occurrence of PSM following curative resection for gastric cancer [15]. These findings suggest that the incidence of PSM after curative gastrectomy may be lower than that after other cancers. Therefore, the clinicopathological features and treatment strategies for PSM after gastrectomy are not well investigated.

There are only five detailed reports of PSM recurrence after curative resection of gastric cancer [3-7] (Table 1). Previous reports demonstrated that PSM mainly arose on the left side, ranging in size from 2.5 to 5 cm. Primary tumors with poor to moderate differentiation were reported from stage I to IIIB. The histological type of the primary tumor and the time from the initial surgery to the appearance of PSM might not correlate with PSM size. Compared with other cases, ours was different in morphology, with widespread longitudinal invasion into skeletal muscle. In addition, all other cases did not exhibit distant metastases and underwent surgical resection. The etiology of PSM is unclear. In the previous report, surgical techniques such as improper direct tumor manipulation and pneumoperitoneum were identified as risk factors for PSM [8]. The non-touch isolation technique might contribute to preventing PSM recurrence; however, it was not sufficient in gastric cancer because PSM recurrence occurred even in an early case [4]. Surgically resected PSM cases remained recurrence-free for at least three months in all cases and for more than three years in some cases [5,6]. One report described recurrence in another part of skeletal muscle in the abdominal wall after PSM excision [6], distinct from the PSM site. These reports indicate that surgical treatment for localized PSM without distant metastasis would be effective for local control.

**Table 1:** Previous reports of port-site metastasis. M: male; NA: not available; pStage: pathological stage; PSM: port-site metastasis *: At 13 months postoperatively, recurrence was noted in the left rectus abdominis muscle. At 35 months postoperatively, another recurrence was noted in the left gluteus muscle; both were solitary recurrences and were surgically resected.

No	First author	Year	Age	Sex	pStage	Primary tumor differentiation	Interval between surgery and PSM appearance	PSM abdominal location	PSM long diameter	Concomitant metastases	PSM treatment	Prognosis after PSM appearance
1	Lee [3]	2007	73	M	ⅢB	Poorly differentiated	12 months	Left-lower	2.5cm	None	Surgical resection/ postoperative chemotherapy	6 months relapse-free
2	Sakurai [4]	2013	58	M	ⅠA	Moderately differentiated	18 months	Left-lower	4.5cm	None	Surgical resection	12 months peritoneal recurrence
3	Kim [5]	2015	78	M	ⅡA	Poorly differentiated	6 months	Right-lower	4cm	None	Surgical resection	50 months relapse-free
4	Fukui [6]	2021	75	M	ⅢA	Moderately differentiated	23 months	Left-umbilical	NA	None	Surgical resection	55months alive*
5	Namikawa [7]	2021	66	M	ⅡA	Poorly differentiated	42 months	Left-upper	5cm	None	Surgical resection/ postoperative chemotherapy	3 months relapse-free
6	Our case	2024	55	M	ⅢA	Poorly differentiated	24 months	Left-upper	13cm	Retro-peritoneal	Chemotherapy/ radiation	14 months dead

The treatment outcome of chemotherapy and radiotherapy for PSM is unknown. For skeletal muscle metastases from gastric cancer occurring distant to port sites, the therapeutic effect of chemotherapy, including molecularly targeted agents, has been limited [16-18]. On the other hand, palliative radiotherapy occasionally results in symptomatic control and downsizing [16,17,19]. In an exceptional case, skeletal muscle metastasis achieved a complete response with chemo-radiotherapy [20]. In our case, despite administering chemotherapy and radiotherapy for skin-infiltrating and inguinal lesions due to PSM progression, the treatment outcome was unsatisfactory in terms of relief from cancer pain and impaired walking.

Previous reports indicate that effective local control could be achieved by surgical intervention for lesions less than 5 cm in maximum diameter, suggesting the potential benefit of early diagnosis and surgical treatment for PSM. A previous case report demonstrated the expansion of PSM from 2 to 4 cm in two months [5]. Furthermore, the onset of PSM varies widely, ranging from six to 42 months. Therefore, early detection of PSM requires not only self-observation of the surgical site and abdominal physical examination but also routine and occasional imaging studies during the standard five-year follow-up period for gastric cancer. Palliative resection should be considered for PSM cases detected early because extensive PSM enlargement can potentially deteriorate the quality of life and ADL.

## Conclusions

PSM, which spreads widely along skeletal muscles, can provoke opioid-resistant cancer pain and impaired walking. Chemotherapy and radiotherapy for PSM are limited in their capacity for local control and symptom management, and palliative resection is most useful for local control. Although PSM recurrence is rare after gastrectomy for gastric cancer, careful surveillance by routine and occasional computed tomography studies based on self-observation and physical examination might help detect it at a resectable size.
